# Maternal exposure to metal components of PM2.5 and low birth weight in New Mexico, USA

**DOI:** 10.21203/rs.3.rs-2666605/v1

**Published:** 2023-03-31

**Authors:** Yanhong Huang, Xi Gong, Lin Liu, Li Luo, Shuguang Leng, Yan Lin

**Affiliations:** The University of New Mexico - Albuquerque: The University of New Mexico; University of New Mexico; University of New Mexico - Albuquerque: The University of New Mexico; University of New Mexico - Albuquerque: The University of New Mexico; University of New Mexico - Albuquerque: The University of New Mexico; University of New Mexico - Albuquerque: The University of New Mexico

**Keywords:** low birth weight (LBW), Air pollution, Exposure assessment, GIS, Particulate matter (PM)

## Abstract

Infants with low birth weight (LBW) are more likely to have health problems than normal weight infants. In studies examining the associations between particulate matter (PM) exposures and LBW, there is a tendency to focus on PM_2.5_ as a whole. However, insufficient information is available regarding the effects of different components of PM_2.5_ on birth weight. This study identified the associations between maternal exposure to 10 metal components of PM_2.5_ and LBW in offspring based on small area (divided by population size) level data in New Mexico, USA, from 2012 to 2016. This study used a pruned feed-forward neural network (pruned-FNN) approach to estimate the annual average exposure index to each metal component in each small area. The linear regression model was employed to examine the association between maternal PM_2.5_ metal exposures and LBW rate in small areas, adjusting for the female percentage and race/ethnicity compositions, marriage status and educational level in the population. An interquartile range increase in maternal exposure to mercury and chromium of PM_2.5_ increased LBW rate by 0.43% (95% confidence interval (CI): 0.18%−0.68%) and 0.63% (95% CI: 0.15%−1.12%), respectively. These findings suggest that maternal exposure to metal components of air pollutants may increase the risk of LBW in offspring. With no similar studies in New Mexico, this study also posed great importance because of a higher LBW rate in New Mexico than the national average. These findings provide critical information to inform further epidemiological, biological, and toxicological studies.

## 1. Introduction

Low birth weight (LBW) refers to an infant who is under 2500 grams (5.5 pounds) at birth (WHO, 2014). LBW is closely associated with adverse health outcomes, such as increased fetal and neonatal mortality and morbidity, stunted growth, and cognitive impairment (WHO, 2019). Besides, it could increase the risks of hearing impairment (Daghistani et al., 2002), cardiovascular disease (Alexander et al., 2014), obesity (Casey et al., 2012), and respiratory distress (Hasmasanu et al., 2018) in children and adults. Moreover, LBW is associated with poorer mental health and well-being than normal-weight infants (Lund et al., 2012). In New Mexico, 9.1% of newborns have been born LBW between 2012 and 2021, which is higher than the national average of 8.2% (NCBI, 2022; U.S. CDC, 2022). Besides, New Mexico is a largely rural state, has racially/ethnically diverse population, and has higher poverty rate (18.4% in 2021) than national rate (11.6% in 2021) (U.S. Census Bureau, 2022b), which makes it a unique study area. Therefore, it is necessary to study LBW in New Mexico for its possible causes.

The major risk factors for LBW of infants include genetics, maternal characteristics and behaviors (mother’s age, physical work during pregnancy, and smoking and drinking status), socioeconomic factors (marriage status, income level, educational level, stress, and domestic violence), and environmental risk factors (maternal exposure to air pollution, water pollution, and soil pollution, etc.) (Gong et al., 2018; Hasmasanu et al., 2018; Hidalgo-Lopezosa et al., 2019; Khan et al., 2016; Mahmoodi et al., 2013; U.S. CDC, 2001; Valero De Bernabé et al., 2004). Many studies have discovered that maternal exposure to air pollutants (including particulate matter (PM)) are associated with LBW in offspring (Ebisu et al., 2014; Liu et al., 2019; Rodríguez-Fernández et al., 2022; Xiao et al., 2018a).

Particulate matter (PM) contains both solid particles and liquid droplets in the air, including PM_10_ and PM_2.5_ (inhalable particles with diameters of less than 10 micrometers and 2.5 micrometers respectively) (U.S. EPA, 2021). Metal particles, such as vanadium, nickel, chromium, copper, and zinc, can be transported by PM_2.5_ (Chen & Lippmann, 2009). In human bodies, most metal elements tend to be bio-accumulated and become toxic. Studies have shown that exposure to metal components of air pollution can adversely affect health outcomes in a variety of systems, such as the respiratory system (Tager et al., 2005), cardiovascular system (Ghio, 2006), nervous system (Ratnaike, 2003), etc. Furthermore, studies have shown that maternal exposure to heavy metals can interfere with fetal development and result in adverse birth outcomes (Bellinger, 2005; Garza et al., 2006; Guo et al., 2022; Schell et al., 2006). Metal particles generated by industrial and manufacturing processes have been continuously emitted into the air in New Mexico. Therefore, metal components of PM_2.5_ that are potentially related to LBW in New Mexico are causing increased concern.

However, in studies examining the associations between maternal exposure to PM_2.5_ and LBW in infants, there is a tendency to investigate PM_2.5_ as a whole (Hung et al., 2022; Sun et al., 2016). Ibrahimou et al. (2014) estimated that exposure to PM_2.5_ during the first trimester and during the entire pregnancy period are associated with LBW and very low birth weight. There is a high magnitude of the associations between maternal exposure to PM_2.5_ and decreasing in birth weight of infants in Shanghai (Xiao et al., 2018b). According to Hao et al. (2016), PM_2.5_ exposure during the entire pregnancy or a specific trimester is significantly associated with LBW in the United States. Only a few studies have focused on specific metal components of PM_2.5_. Maternal exposure to PM_2.5_ components such as vanadium, manganese, zinc, and copper were associated with decrease in birth weight in California (Basu et al., 2014; Pedersen et al., 2016). Besides, maternal exposure to chromium, cobalt, and lead were significantly associated with adverse birth outcomes in Portland, Oregon (Comess et al., 2021). In addition, maternal exposure to mercury was found to have significantly higher odds of LBW in offspring in Texas, USA (Gong et al., 2018). However, the impacts of maternal exposure to each metal component of PM_2.5_ to LBW in offspring haven’t been studied in New Mexico.

This study intends to investigate the associations between maternal exposure to metal components of PM_2.5_ during pregnancy and LBW in offspring in the state of New Mexico from 2012 to 2016. This study covered 10 metal components of PM_2.5_ and analyzed the associations among the small areas of New Mexico, which consist of 108 regions with similar populations. As a first study in New Mexico, this study fills a literature gap by focusing on different PM_2.5_ metal components rather than considering them as a whole.

## 2. Study Area, Data, And Methods

### 2.1 Study area

**Figure 1:**
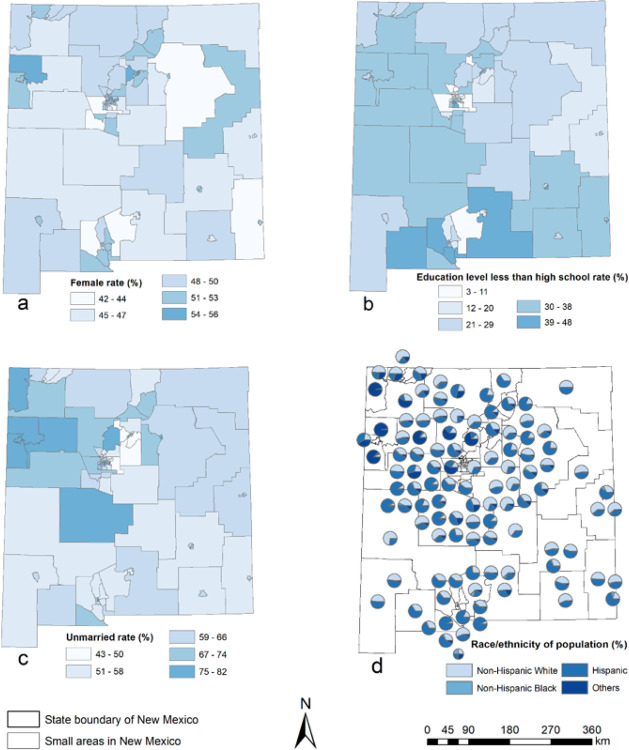
Five-year-average basic birth characteristics (a: percentage of female population; b: percentage of population with less than high school education level; c: percentage of unmarried population; d: race/ethnicity composition of the population) in small areas, New Mexico, 2012–2016.

The study area is the state of New Mexico (NM) in the Southwestern United States. With a total area of 314,914 km^2^, NM has just over 2.1 million residents of racially/ethnically diverse population. There are 33 counties in New Mexico, but more than one-third population lives in Bernalillo County. Reporting health data on county level is problematic in New Mexico because of the large differences in county population sizes. To improve the efficiency in production of reports and accumulation data across public health datasets on community-level, the New Mexico Department of Health (NMDOH) built small areas on population size rather than land area (NM-IBIS, 2017). In this study, we chose the 108 small areas in New Mexico (Fig. 1) as the spatial unit for further analysis.

### 2.2 Data and methods

We used the pruned feed-forward neural network (pruned-FNN) method (Gong et al., 2022 PREPRINT) to estimate the annual average maternal residential exposure to each metal component of PM_2.5_ in each small area during 2012–2016, then applied linear regression to identify associations between maternal PM_2.5_ metal exposures and LBW rate in small areas after adjusting for potential confounders. Details about data and methods are as follows:

#### 2.2.1 Exposure assessment

This study used a custom-designed pruned feed-forward neural network (pruned-FNN) model (Gong et al., 2022 PREPRINT) to simulate the complex and non-linear relationships from air pollution emission to individual exposure. The exposure assessment model that can strike a balance between accuracy, complexity, and usability, which is an appropriate and effective approach for exposure assessment that covers a large geographic area over a long period of time. Emission time and emission rate of air pollutants, terrain factors, meteorological conditions, and proximity measurements are used as input variables of this model; monitoring data is considered as the ground truth to train, calibrate, and cross-validate the pruned-FNN (Gong et al., 2022 PREPRINT).

Equation (1) is used to calculate the pollution exposure indexes (PEIs) of a given chemical *k* at a given location i:

1 $$PEI_{i,k}=\sum_{j=1}^{n}g_{j}\left(T_{i},H_{i},EM_{j,k},D_{i,j},ED_{i,j},W_{i,j}\right)$$

The ***PEI***_*i,k*_ is the predicted exposure index of a chemical *k* at the location *i*, and *j* represents one of the *n* total sources of emission (factories). The *g*_*j*_ is the contribution of PEI at location *i* from a single emission source j. It is generated by the complex dispersion of air pollutants, which can be represented by six independent variables. These variables are ***T***_*i*_ (the temperature at the location i), ***H***_*i*_ (the humidity at the location *i*), ***EM***_*j,k*_ (the sum of fugitive and stack emissions of chemical *k* from emission source *j*), ***D***_*i,j*_ (the distance from location *i* to the emission source *j*), ***ED***_*i,j*_ (the elevation difference between emission source *j* and location *i*), and ***W***_*i,j*_ (the calculated wind index between emission source *j* and location *i*).***PEI***_*i,k*_ is the sum of all *g*_*j*_ (*j* = 1,2, …, n). More details of the model design can be found in (Gong et al., 2022 PREPRINT).

The emissions datasets come from the Toxics Release Inventory (TRI) Program of the U.S. EPA, which requires the facilities to report the emission information (locations of facilities, types of chemicals, and estimated quantity of chemicals) annually in United States (U.S. EPA, 2022). There were 369 industrial facilities which have reported air emissions in New Mexico and its surrounding area during 2012–2016 (Fig. 1). We collected the sum of fugitive and stack emission data of those facilities, where 139 air pollutants were emitted into the air in New Mexico and its surrounding area during this time period. The monitoring data is collected from Air Quality System (AQS) DataMart of the U.S. EPA (U.S. EPA, 2018). This study collected the monitoring data of 255 air pollutants from 63 monitoring sites in New Mexico from 2012 to 2016 (Fig. 1). We selected the 10 metal components of PM_2.5_ (barium, chromium, cobalt, copper, lead, manganese, nickel, mercury, vanadium, and zinc) that are shared between the emission and monitoring datasets for training process of the pruned-FNN model. Besides, we also used the climate datasets (temperature, humidity, and wind data) from North American Regional Reanalysis (NARR, 2022) and corresponding distance, elevation differences, slope, and aspect for emission and monitoring sites calculated based on the 30-meter DEM data from United States Geological Survey (USGS, 2019) to predict the air pollution exposure intensity.

After training, calibrating, and 10-fold cross-validating, the pruned-FNN model predictions have high and stable correlations with monitoring data (Gong et al., 2022 PREPRINT). We applied this model to estimate the annual average exposure in each small area by using monitoring data as reference. We built a grid with spatial resolution at 4050m*4050m covering the entire state to match the spatial scales of the climate data. Then we used the trained model to predict the annual air pollution exposure intensity in each grid for the 10 metal components of PM_2.5_ during 2012–2016. For this study, we assumed that the population is evenly distributed within each small area, so the annual average exposure intensity of all grids in a small area is used to represent the maternal PM_2.5_ metal exposure intensity in the small area in the corresponding year.

#### 2.2.2 Identification of potential risk factors

The birth data of New Mexico during 2012–2016 was obtained from New Mexico’s Indicator-Based Information System (NM-IBIS, 2022). The NM-IBIS calculates the LBW rate at different aggregate levels (e.g., state, county, and small area) based on birth certificate data from the New Mexico Department of Health (NMDOH) Bureau of Vital Records and Health Statistics (BVRHS). This study used the average LBW rate at the small area level during 2012–2016 in New Mexico for further analysis, the range of which is 6.2–12.9% (Fig. 1).

Air pollution is not the only risk factor for LBW, social economics factors and the mother’s characteristics (age, education and marital status) are also related to LBW in offspring (Hidalgo-Lopezosa et al., 2019; Ojha, 2015). To reduce confounding effects, we need to adjust the air-pollution-LBW association with these factors. We collected the datasets regarding potential confounders including gender, education level, race/ethnicity, and marital status of the population at census tract level from the United States Census Bureau during 2012–2016 (U.S. Census Bureau, 2022a). To be consistent with the spatial scale of birth data, areal interpolations are used to reaggregate the data from census tract level to small area level.

Univariable and multivariable linear regression models were used to identify the associations between maternal exposure to each of the 10 PM_2.5_ metal components and LBW rate in New Mexico from 2012 to 2016. We calculated the five-year-average maternal exposure to each metal component in each small area to match the temporal scale of the LBW rate. These calculated exposure values were the independent variable and the LBW rate in each small area was the dependent variable. We also adjusted for the female percentage and race/ethnicity compositions in the population, as well as marriage status and educational level of the population in the regression to minimize confounding. Finally, the association is measured through the LBW rate changes per inter-quartile range increase (IQR) in exposure intensity of each of the 10 metal components of PM_2.5_. The Benjamini-Hochberg procedure was used to adjust for multiple comparisons.

## 3. Results

Figure 1 shows the ve-year-average basic characteristics of births at small area level in New Mexico from 2012 to 2016. There are approximately 50% females in each small area (Fig. 1a). All the small areas have more than half of the population with a high school diploma or higher; ve small areas in Southern New Mexico have more than one third of residents who have not finished the high school education (Fig. 1b). New Mexico’s northwest region has the higher percentage of unmarried residents than other area (Fig. 1c). Most small areas are racial/ethnically diverse, except for the northwestern small areas where Navajo Nation located (Fig. 1d).

Table 1 shows the associations between exposure to each of the 10 metal components of PM_2.5_ and LBW after adjusting for covariates. Maternal exposure to two metal components of PM_2.5_ (mercury and chromium) have significant positive associations (adjusted *p*-value < 0.05) with LBW rate in offspring after multiple comparison correction. LBW rate increases 0.43% (95% confidence interval (CI): 0.18%−0.68%) and 0.63% (95% CI: 0.15%−1.12%) per interquartile range increase in maternal exposure to mercury and chromium in PM_2.5_ respectively. Besides, maternal exposure to nickel, lead, and copper in PM_2.5_ can also increase LBW rate by 0.35% (95% CI: 0.08%−0.62%), 0.32% (95% CI: 0.05%−0.60%), and 0.50% (95% CI: 0.07%−0.93%) respectively, but the associations became insignificant after multiple comparison corrections.

## 4. Discussion

This study assessed maternal exposure to 10 metal components of PM_2.5_ in New Mexico from 2012 to 2016 based on a machine learning method (pruned-FNN), and calculated associations between maternal exposure to these components and LBW rate. We identified that maternal exposure to mercury and chromium in PM_2.5_ shows significant positive associations with LBW rate.

Adverse health outcomes of exposure to chromium and mercury have been well documented. As a global pollutant, exposure to mercury is associated with delayed neurodevelopment in children and metabolic syndrome in adulthood (Ha et al., 2017). It has been identified that the inhalation of chromium is significantly associated with lung and nasal cancer (Paustenbach et al., 2003). Paustenbach et al. (2003) found that exposure to chromium in PM is associated with inhalation cancer risk in the most highly industrialized region of Poland. Even low level exposure to mercury had the impacts on nerve system and immune system of people (Holmes et al., 2009). Moreover, the relationships between maternal exposure to these two chemicals (mercury, chromium) and the LBW have also been identified by other researchers in other study areas. 2.5% increase in LBW rate was associated with an inter-quartile range increase in chromium in PM_0.1_ in Los Angeles County, California, 2001–2008 (Laurent et al., 2014). In a case-control study in China, Xia et al. (2016) found a significant correlation between LBW and high maternal urinary chromium levels. Gong et al., (2018) estimated associations between maternal residential exposure to 14 industrial air pollutants during pregnancy and LBW in offspring in Texas from 1996 to 2008 and found that mercury exposure is positively associated with LBW (aOR 1.04, 95% CI 1.02, 1.07). Besides, it has been identified that there is a significant negative association between maternal exposure to mercury during the first and second trimesters of gestation and birth weight in offspring in Tokyo during 2010 to 2012 (Vigeh et al., 2018). These findings are consistent with the results of this study.

**Figure 2:**
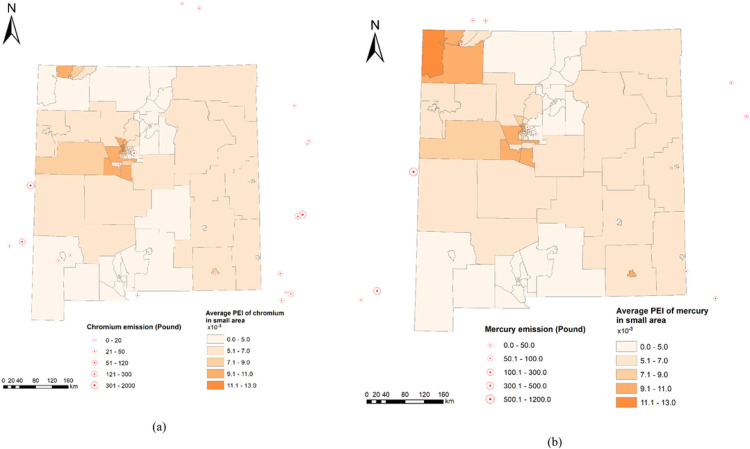
Geographic distribution of industrial facilities that emitted (a) chromium and (b) mercury and average pollution exposure index (PEI) of small areas in New Mexico and surrounding area during 2012–2016.

From 2012 to 2016, industrial facilities in central and southwest of New Mexico and surrounding area emitted the chromium and mercury to the air (Fig. 2). Central and northwestern small areas of New Mexico have higher average PEI of mercury and chromium than other small areas (Fig. 2). Future studies should focus on these areas to study other health effects of the two metal components of PM_2.5_.

**Figure 3:**
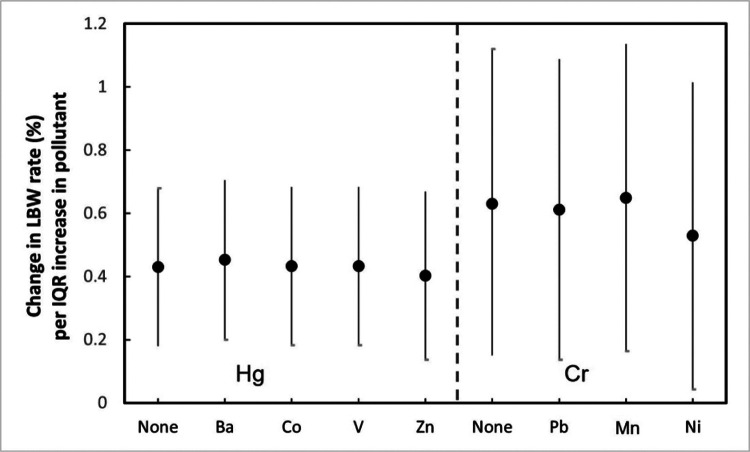
Change in LBW rate per IQR increase in maternal exposure to pollutant, for single and two-pollutant linear models. The point reflects the central estimate, the vertical line represents the 95% confidence interval.

Figure 3 Change in LBW rate per IQR increase in maternal exposure to pollutant, for single and two-pollutant linear models. The point reflects the central estimate, the vertical line represents the 95% confidence interval.

For each of the two identified metal components, we used the exposure to other metal components to adjust for the results of the linear regression. We only selected the metal pairs which were uncorrelated (Pearson correlation coefficient < 0.5) in two pollutant linear models. Figure 3 shows the results after adjustment. There are small variations of LBW rate changes per IQR increase maternal exposure in mercury and chromium when other metal components of PM_2.5_ are considered. The relationships between maternal exposure to mercury and chromium and LBW rate are robust.

There are several limitations in this study. First, the air emission data we used only included the TRI industrial facilities, which are stationary sources of emissions. Other types of emission sources such as linear, areal, and mobile sources were not included in this study. In future research, we can incorporate other emission sources such as traffic air pollution sources, uranium and coral mine sites, and mobile sources to get a more accurate result. The pruned-FNN model has the fault tolerance property of the neural network, which means that it can deal with the incompleteness and uncertainty of the input data to a certain extent.

Second, we used the monitoring data as ground truth data in the training process for pruned-FNN model to estimate the air pollution exposure intensities. Therefore, we can only train pruned FNN models with chemicals that are shared by emission datasets from industrial facilities and monitoring recordings, which limits the model’s coverage of air pollutant type. As a result, a monitoring network that covers a wider geographical area and more pollutants in New Mexico is necessary. Most of the monitoring sites are not evenly distributed, with most sites located in urban areas. In suburban and rural areas, we may get less accurate results of exposure than in urban areas because there are insufficient training samples in those areas. To obtain more accurate estimates of air pollution exposure, further studies should be conducted in urban areas.

Third, we included emission data from nearby states such as Arizona, Texas, and Colorado to eliminate edge effects. However, we did not include emission data from Mexico due to the fact that the data sources were inaccessible, which might lead to less accurate results. It is important to include data from Mexico in order to eliminate the edge effect and make the results more accurate in the future studies.

Finally, the birth data were aggregated rather than considered individually and we assumed that the population are evenly distributed in each small are in this study. There are only 108 aggregated small areas in the state of New Mexico. Small areas were selected as the geographic scale as the compromise to inaccessible data for individual participants in this study. In addition, the birth data we used is 5-year average, we need to aggregate the exposure data to match the birth data’s temporal scale (5-year average) for analysis. Additionally, the covariates used to adjust regression results were also aggregated into small areas in order to align both with exposure data and birth data. We could only get a summarized statistics for each area, which is less accurate than individual results, but the datasets of LBW and covariates we used are public available, and it is good for exploratory analysis. In the future, we can consider requesting individual level birth data for further analysis.

## 5. Conclusion

This study investigated the possible relationship between maternal exposure to metal components of PM_2.5_ during pregnancy and LBW in offspring using data from New Mexico during 2012–2016. The main contribution of this study is that it covered 10 metal components of PM_2.5_ in air-pollution-LBW association explorations. It is also the first study in New Mexico on maternal PM_2.5_ metal exposures as LBW risk factors, which is crucial because of the higher LBW rate than national average. Study results suggested that maternal exposure to mercury and chromium in PM_2.5_ are positively associated with LBW rates in offspring. Further epidemiological, biological, and toxicological studies are recommended to verify these findings.

## Figures and Tables

**Table 1:** Maternal exposure to 10 metal components of PM_2.5_ and LBW in offspring, New Mexico, 2012–2016.

Metal components in PM_2.5_	LBW rate change per IQR increase in pollutant exposure	95% CI	*p*-value	FDR-adjusted *p*-value	IQR (×10^−3^)
Mercury (Hg)	0.43%	0.18% – 0.68%	<0.001*	0.005	0.307
Nickel (Ni)	0.35%	0.08% – 0.62%	0.011	0.010	0.731
Chromium (Cr)	0.63%	0.15% – 1.12%	0.011*	0.015	1.729
Lead (Pb)	0.32%	0.05% – 0.60%	0.022	0.020	0.961
Copper (Cu)	0.50%	0.07% – 0.93%	0.025	0.025	1.390
Zinc (Zn)	0.36%	−0.06% – 0.78%	0.150	0.030	1.673
Cobalt (Co)	0.24%	−0.20% – 0.68%	0.374	0.035	0.035
Vanadium (V)	0.24%	−0.21 % – 0.69%	0.388	0.040	0.238
Manganese (Mn)	0.12%	−0.20% – 0.44%	0.530	0.045	0.867
Barium (Ba)	0.12%	−0.25% – 0.49%	0.592	0.050	3.355

* Statistically significant after Benjamini-Hochberg procedure for multiple comparisons (false discovery rate: 0.05).

## Data Availability

The data that support the findings of this study are available from the corresponding author upon reasonable request.
